# Innovation in developing isolated coastal villages into sustainable marine tourism villages in West Sumatra, Indonesia: adaptive capacity, social capital, and transformation

**DOI:** 10.3389/fsoc.2026.1754562

**Published:** 2026-03-03

**Authors:** Delmira Syafrini, Lucky Zamzami, Erna Herawati, Lia Amelia, Hanafi Saputra, Nora Susilawati, Bunga Dinda Permata, Chikita Arone

**Affiliations:** 1 Department of Sociology, Faculty of Social Science, Universitas Negeri Padang, Padang, West Sumatra, Indonesia; 2 Department of Anthropology, Faculty of Social and Political Science, Universitas Andalas, Padang, West Sumatra, Indonesia; 3 Department of Anthropology, Faculty of Social and Political Science, Universitas Padjadjaran, Padang, West Sumatra, Indonesia

**Keywords:** isolated coastal areas, marine tourism, social capital, social transformation, sustainable development, tourism fishermen

## Abstract

This study is focused on explaining how social capital and adaptive capacity influenced the successful development of isolated coastal villages into sustainable marine tourism villages in Mandeh Integrated Marine Tourism Area (KWBT), West Sumatra, Indonesia. The region is among the few isolated areas that had successfully developed into a marine tourism village in a relatively short time, roughly less than 10 years. Therefore, a qualitative case-study approach, was adopted with data gathered through observation and interviews with 34 stakeholders, including local residents and tourists. The results showed that marine tourism had offered new opportunities to the local community, benefiting the economy including social and cultural aspects. However, the success of the development was driven by internal strengths rooted in the community, such as the adaptive capacity and social capital, with most residents being traditional fishermen. This includes: (1) Family values and solidarity as bonding social capital, (2) trust as bridging social capital, which underpins participation and cooperation in village development, and (3) collaboration with migrants and stakeholders as linking social capital. The novelty of this study lies in strengthening the analysis from sociological and anthropological perspectives through the integration of Structural-Functional Theory, Adaptive Capacity, and Social Capital to explain the roles of adaptive capacity and social capital as key defensive mechanisms enabling fishing communities to adjust to change. The results provided important practical and academic implications, as it was recommended that social capital, adaptability, and community cultural values were crucial factors to be considered in achieving sustainable development in coastal areas.

## 1 Introduction

The development of coastal villages through marine tourism programs is the main focus on many small and isolated islands in various countries. This is due to the popularity of marine tourism as a travel option worldwide, specifically on remote islands (Xie et al., 2013; Fonseca et al., 2014; Kinseng et al., 2018). The trend is connected to shifting global tourism preferences, which pursue new, unique, and memorable experiences (Ning, 2017; Atasoy, 2021). As a result, small islands with pristine natural landscapes, peaceful environments, and rich local cultures have become major draws for tourists (Croes et al., 2013).

The advancement of marine tourism in small islands and isolated coastal areas attracts tourists as well as promotes the growth of this sector. Numerous studies have shown that tourism tended to improve local economic (Hampton and Jeyacheya, 2015; Praptiwi et al., 2021; Fernandez-Abila et al., 2024) and regional development in previously underdeveloped small islands and coastal areas (Sharpley and Ussi, 2014).

Based on this perspective, the development of marine tourism had been prioritized in Indonesia over the past decade. A typical example of an area that has focused on and successfully developed marine tourism is Pesisir Selatan Regency, West Sumatra. This also included the main tourist attraction, Mandeh Integrated Marine Tourism Area (KWBT). The attraction comprised dozens of islands covering 18,000 hectares, featuring stunning scenery with natural green hills, sea, bays, and pristine beaches (Alhadi et al., 2023). However, before the development as tourist attraction, this area was an isolated village with no land access and could only be reached by sea. Mandeh region covers three *nagari* (a traditional administrative unit in West Sumatra, Indonesia, based on customary law) and seven additional villages, with a total population of 10,867 residents. Most of residents were fishermen, livestock raisers, or farmers. In 2015, the development of marine tourism caused a major change in the area, as a land route was opened, making it a popular destination for tourists. The previously isolated village is among Indonesian leading marine tourism spots, drawing roughly 2 million tourists each year (BPS Kabupaten Pesisir Selatan, 2025), and earning the nickname a hidden paradise (Fatimah and Ramadhan, 2019).

Mandeh exemplifies a handful of isolated villages that have changed quite rapidly. In merely 10 years, the village had witnessed major shifts, specifically in the socio-economic life of its residents. The people who originally depended on traditional fishing, currently work as tourism fishermen. In addition to not losing the identity as fishermen, the people actively participated as tourism service providers and earned significant profits from this sector. During the holiday season or on weekends, the fishermen earned up to IDR 2–3 million per day, an income that was made as traditional fishermen.

The most interesting aspect was that this rapid transformation occurred due to the strong internal values of the local community, expressed in collective beliefs maintained for generations to survive. The values formed the basis for adaptability and social capital that drove collective efforts to support the development of the village. Although this transformation has the potential to generate social dynamics and conflicts, such as unequal access to tourism opportunities, competition among local actors, shifts in power relations, and the potential marginalization of groups lacking economic capital or social networks these risks are relatively manageable and can be minimized due to strong norms of mutual cooperation, social trust, and local deliberative mechanisms that function as spaces for collective negotiation and problem-solving. This deeply rooted social capital enables the community to maintain social cohesion while ensuring that the transformation process remains inclusive and sustainable.

Based on the problems above, the social capital and adaptive capacity of Mandeh fishing community were the main focus of this study. The study aims to explain how these strengths have facilitated the transformation from isolated coastal villages into marine tourism villages in the Kawasan Wisata Terpadu Mandeh (KWBT), West Sumatra, Indonesia. This led to the exploration of several specific areas, including: (1) how the adaptive capacity of the local community served as the main driver for innovation in developing marine tourism villages, and (2) the diverse forms of social capital that promoted the successful transformation of fishing villages into sustainable marine tourism villages.

In many areas, transformation due to marine tourism development occurred slowly, specifically regarding social change in the local community, where fishermen were often marginalized. However, in Mandeh area, the transformation happened relatively quickly, driven by the strengths of the local community’s social and cultural structures. Prior studies on the social capital and adaptive capacity of coastal communities in responding to transformations caused by tourism development remained limited. The previous analyses on marine tourism briefly discussed the development process and its impact on the economy (Tegar and Saut Gurning, 2018; Srinivasan et al., 2022; Reddy and Sailesh, 2024). This included challenges faced (Fonseca et al., 2014; Papageorgiou, 2016; Kinseng et al., 2018), marine tourism and environmental conservation (Apps et al., 2018; Mangubhai et al., 2019; Hofman et al., 2022; Misra and Miller, 2022), alongside sustainable tourism development in coastal areas (Wang and Zhang, 2019; Cong and Van Chi, 2021).

Considering that previous studies had explored marine tourism in the broader scope of tourism development, it paid slight attention to the socio-cultural aspects of the community, such as social capital and adaptability, in the context of sustainability. These socio-cultural factors play a critical role in the evaluation of coastal area transformation. However, its social and cultural traits tend to significantly influence development success, and in some cases hinder growth.

The novelty and implications of this study was strengthened by offering a new perspective on analyzing the results from a sociological and anthropological view. Additionally, a combination of Structural-Functional Theory (Parsons, 1961), Adaptive Capacity (Folke et al., 2003), and Social Capital (Coleman, 1990; Putnam, 2015) were adopted to explore how adaptive capacity and social capital served as defenses for communities facing crises. Changes in environmental conditions did not destroy or marginalize the factors, rather both made the community more resilient and promoted growth. This study further contributed to policy-making for sustainable development in coastal areas by integrating the local community’s socio-cultural aspects into the formulated approach. In addition, the integration ensured that the local community, specifically the fishermen, were no longer marginalized in tourism development.

## 2 Literature review

### 2.1 Marine tourism and sustainable development in coastal villages

Marine tourism is a type of travel that outlines the sea and coastal ecosystems as its main attractions. According to Mark Orams (2002), it includes all activities that occur in marine settings, beaches, and small islands, whether focused on recreation, sports, education, social-cultural tasks, or conservation. In respect to recreation, marine tourism centers on entertainment and enjoyment, namely swimming, snorkeling, diving, and surfing, which provide direct experiences of the sea’s beauty (Orams, 2002).

Conservation marine tourism focused on the protection and preservation of the ecosystems, such as mangrove, coral reef transplantation, and marine wildlife conservation (Wilson and Tisdell, 2003; Apps et al., 2018). Educational marine tourism centered on learning and related activities, namely school trips to conservation areas, observation of marine life, and tourists’ participation in coastal ecosystem studies (Hall, 2001). Meanwhile, socio-cultural marine tourism explored marine values of the local community, including sea festivals, traditional fishing ceremonies, history tours, and seafood-based culinary experiences (Khakzad and Griffith, 2016; Kong, 2024).

Marine tourism is the main tool for developing coastal villages, due to the promotion and preservation of local economic growth and ecosystem health (Tegar and Saut Gurning, 2018; Wang and Zhang, 2019). The transformation process supports the principles of sustainable development, which combines economic, social, and environmental factors (Wang and Zhang, 2019; Booth et al., 2022). Recent studies reported that marine tourism greatly aided sustainable development when managed inclusively, alongside community participation (Tegar and Saut Gurning, 2018; Booth et al., 2022; Rahman et al., 2022).

Based on an economic perspective, marine tourism functioned as an alternative income source for coastal communities, creating jobs and improving local economic diversification (Wang and Zhang, 2019; Srinivasan et al., 2022). Regarding a social perspective, the success of the development process depended on active participation from the local community, because it is believed to enhance quality of life and social cohesion (Rahman et al., 2022). In line with an environmental perspective, marine tourism reportedly improved resource management practices, directly protecting the diverse ecosystems (Apps et al., 2018; Zamzami, 2021; Booth et al., 2022).

The development of marine tourism in coastal villages is part of a comprehensive sustainable advancement strategy. Meanwhile, coastal villages have a significant opportunity to achieve development transformation, when tourism is designed to be community-based, supported by strong social capital, and oriented towards ecological protection. This transformation contributes to improving community welfare, preserving the marine environment, strengthening marine cultural identity, as well as ensuring the sustainability for both current and future generations.

### 2.2 Social capital and adaptive capacity as driving factors for the development of marine tourism villages

Social capital is essential for achieving the development process (Mura and Tavakoli, 2014; Sunkar et al., 2016; Soulard et al., 2018), playing a strategic role in integrating the three core pillars of sustainable marine tourism. This consisted of economic factors, environmental sustainability, and the socio-cultural well-being of the local community. In this context, social capital refers to the resources held by the local community, namely social solidarity, networks, and commitment, participation, and trust, which formed the basis of the collective action to promote tourism development (Sunkar et al., 2016; Nunkoo, 2017).

The concept of social capital has garnered significant attention from experts in sociology. According to Putnam (2015), it is defined as networks, norms, and trust that enabled coordination and cooperation to achieve shared benefits. This concept is also described as the relationship between individuals, including a social asset that enhances community cohesion, and participation, as well as promoted collaboration among actors during development. Meanwhile, James S. Coleman (1990) described it as a resource for achieving set objectives, namely values, norms, rules, and trust among community members which aided the collaboration process.

This non-material resource, develops from networks of social relationships, norms, values, and levels of trust among individuals and groups, enabling cooperation to reach common goals (Putnam, 2015). Social capital focuses on interpersonal relationships, including the bonds in the community (bonding social capital), connections between different groups (bridging social capital), and relationships with broader institutions (linking social capital) (Cofré-Bravo et al., 2019). The concept promotes coordination and collaboration, fosters social solidarity, as well as incited awareness and collective action in the community to engage in tourism development (Soulard et al., 2018).

The understanding of social capital helps refine the analysis of marine tourism by illustrating how the local community collectively acts in the context of innovative development in isolated villages. Putnam and Coleman’s insights served as the foundation for the analysis, reinforcing the idea that support from various stakeholders, local community participation, and collaboration formed social capital that intensified the development of isolated villages into sustainable marine tourism destinations (Loulanskia and Loulanski, 2011).

The theoretical framework was further enhanced by the adoption of social capital theory supported by Parsons’ (1961) Structural-Functional and Carl von Weizsäcker’s Adaptive Capacity Theories. In addition, these two theories offered a more comprehensive understanding of how coastal communities responded to changes, pressures, and threats from both internal and external sources by using respective existing resources and social capital.

In the framework of structural functional theory, the community is perceived as a social system that can only endure change if it can perform the following four core functions Adaptation, Goal attainment, Integration, and Latency. The study by Parsons reported that these functions were referred to as AGIL scheme (Schwandt, 2010). Meanwhile, Folke et al. (2003)Adaptive Capacity Theory outlined the importance of social learning, innovation, flexibility, transformational capacity, and collaborative networks in dealing with uncertainty and dynamic change.

Adaptation (A) function described the importance of the social system’s ability to adapt to its environment, which conformed with the adaptive capacity concept in social-ecological systems. Additionally, Goal Attainment (G) function is related to the capacity of the social system to identify shared objectives and organize resources for sustainable achievement. Integration (I) function stressed the importance of maintaining social cohesion, enabling the system to remain resilient in the face of change. This supported the assumptions of adaptive capacity theory, which described social integration as a significant factor in a community’s ability to resolve conflicts and manage risks (Cinner et al., 2018). Meanwhile, Latency/Pattern Maintenance (L) function corresponded with the idea of adaptive capacity, underscoring the significance of preserving cultural values, local knowledge, and community identity as long-term adaptive resources.

In the context of tourism development in coastal areas, local knowledge of marine cycles, fishing zones, traditional conservation practices, and related customs was part of the cultural resilience. This enabled the adaptation of the community to changes brought about by marine tourism (Pretty and Ward, 2001; Hwang and Stewart, 2017). The combination of social capital, structural-functional, and adaptive capacity theories enhanced the analysis of the results. It also offered new theoretical and practical insights, such as the success of marine tourism village development depended on economic or infrastructural factors. The process included the social system’s ability to adapt, work toward shared goals, maintain cohesion, and protect cultural values when changes occurred due to marine tourism growth. Additionally, social capital formed the foundation connecting the numerous functions, making it a crucial element in transforming isolated villages into a sustainable marine tourism community (Henocque, 2013).

## 3 Method

A qualitative case study approach was adopted because it supported the following objective: to explain how the adaptive capacity and social capital found in coastal communities could be significant drivers of successful development, even transforming isolated villages into visited tourist destinations. This qualitative approach, enabled the thorough exploration of village transformation and marine tourism development, alongside the various forms of social capital, adaptation process, participation of stakeholders and the local community in the entire process.

This study was conducted from May to August 2025, and data collected through participant observation, in-depth interviews, and documentary analysis. Non-participant observation was conducted through direct engagement with members of the local community, particularly fishers, to assess participation in tourism development. In this approach, the researcher assumed the role of an observer who was not directly involved in community activities, while systematically recording interactions, patterns of participation, and social dynamics emerging in the context of tourism-related activities. Observations were carried out across multiple settings, including households, tourism sites, and other tourism-support facilities. The observation procedures included the selection of observation sites and time periods, the use of structured field notes, and the triangulation of observational data with interview materials and documentary sources. This approach enabled a comprehensive assessment of community participation and the impacts of tourism on local social life.

In-depth interviews were held with 34 actors and stakeholders of marine tourism development activities. Informants were selected using purposive sampling, with inclusion criteria aligned with the research objectives. The informants comprised local government officials, tourism office personnel, academics, fishers, community leaders, managers, tour guides, local residents, and tourists. Unstructured interviews were conducted to elicit in-depth and candid data, with the aim of exploring the various forms of social capital derived from collective community values that drive the transformation of villages into tourism destinations. The results were enriched by ensuring the results from interviews and observations were supported by documentation from numerous agencies directly associated with tourism development. This consisted of tourism and culture, alongside marine and fisheries offices, including agencies in each *nagari*.

The results of interviews and observations were transcribed, categorized, and compared with observational and secondary data. Regarding this perspective, data were analyzed using an interactive model across the diverse stages, namely reduction, presentation, and verification (Denzin and Lincoln, 2009). In the initial stage, information were collected through interviews, observations, and documentation on different tourism policies. The processing phase entailed the use of selection, coding, simplification, and transformation. Subsequently, the outcome were organized into a collection of categorized and synthesized information, presented in a narrative form and supported by images, tables, and charts. Data verification and drawing of conclusions were carried out in line with the processes of reduction presentation, and interpretation.

## 4 Findings

The transformation of Mandeh from an isolated area into a marine tourism destination in less than 10 years was a revolution that led to progressive social change. Tourism transformed the area into an attractive destination and also led to significant changes in the lives of the isolated community. Moreover, the success of this development was because of the internal resources of Mandeh community. These included adaptive capacity and social capital derived from shared values, which drove collective action to jointly develop the village for survival and the sustainability of future generations. The present section explained how the internal forces drove the transformation of Mandeh area from an isolated village to a marine tourism destination.

### 4.1 Adaptive capacity of local community as a driver of transformation of isolated coastal villages into sustainable marine tourism villages

Mandeh KWBT has been a leading marine tourism destination in West Sumatra since 2016. The area was visited by approximately 2 million tourists annually, leading to the rapid development (BPS Kabupaten Pesisir Selatan, 2025). Before 2015, the villages in this tourist area were isolated and not accessible by land. Road access was quite limited, with innumerable underdeveloped infrastructure, with the community’s economic activities depending on traditional fisheries and seafood (Fatimah and Ramadhan, 2019). These conditions made the area less known, despite the beautiful natural potential, specifically the cluster of islands, bays, and rich, diverse marine biodiversity.

Mandeh KWBT, consisted of 3 *nagari*, totaling 7 villages. However, an isolated community that had become the center for tourism development was Mandeh Village. Prior to 2016, the fishing village, accommodated 10,867 residents, that heavily depended on marine and forest products for its livelihood (BPS Kabupaten Pesisir Selatan, 2025). The village was inaccessible by land, and the only access was by sea, through fishing boats. This situation caused the local residents to face difficulty accessing the facilities and services necessary for respective livelihoods, including education and healthcare. Subsequently, the village was considered underdeveloped, with low welfare levels.

**Figure 1:**
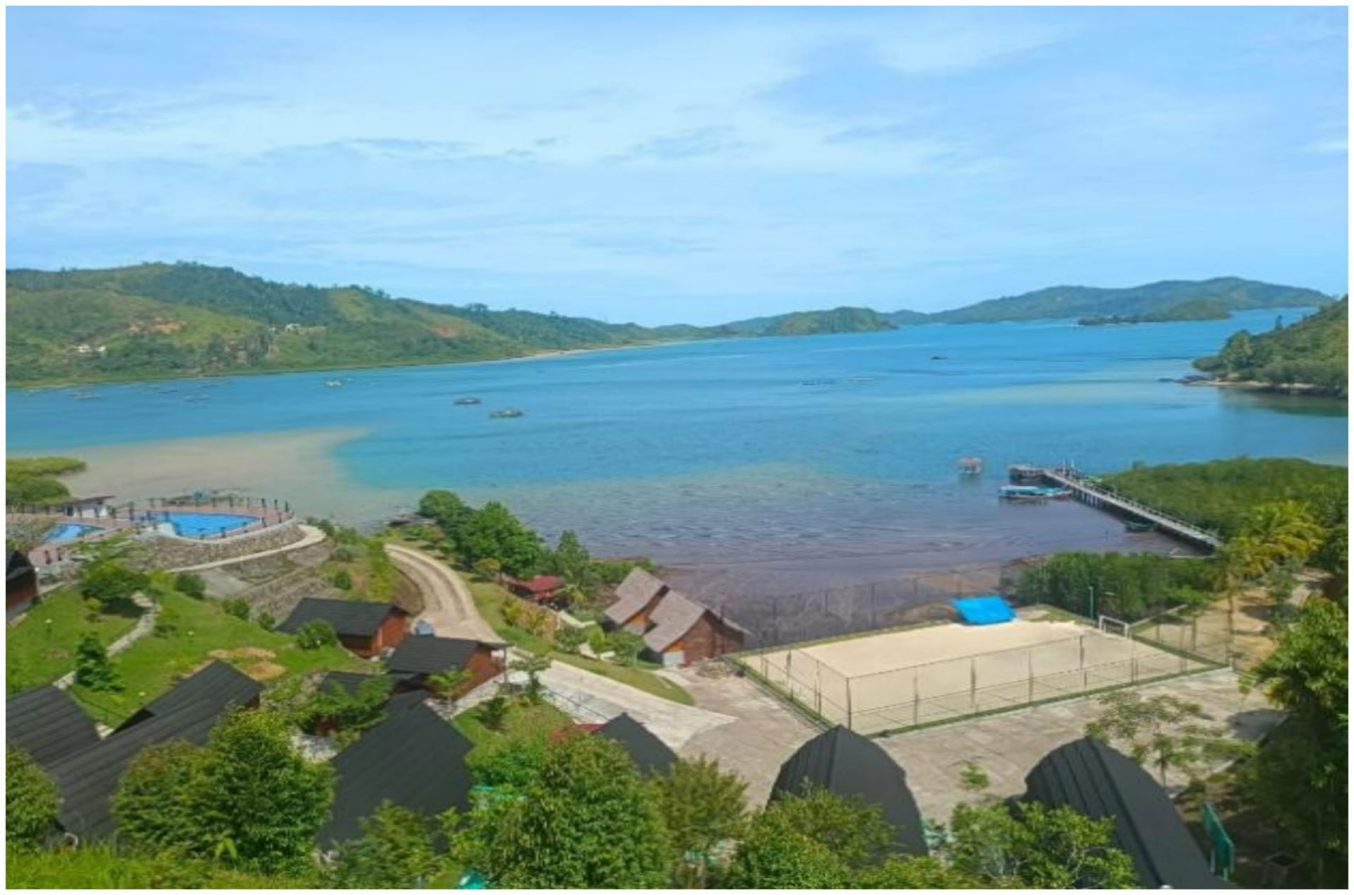
View of the Mandeh Integrated Tourism Area (KWBT Mandeh).

The transformation of the village started in 2019 when the road was constructed and launched. This supported the central and regional government’s policy of designating the region as a National Strategic Tourism Area (KSPN). The main development milestone was the completion of a 41.08-mile access road linking various parts of Padang City and Pesisir Selatan Regency. The construction process began in 2016–2017 by the Ministry of Public Works and Housing (PUPR) and was finished in 2018 (Biro Komunikasi Publik Kementrian PUPR, 2019; Figure 1).

The opening of this land route marked the start of the transformation process. Mandeh area was no longer isolated, as it was visited by millions of people. The development continued with ongoing innovations. The progress was achieved by utilizing local resources and engaging the community. In Mandeh, the local community played a major role in tourism development. However, in other areas, external investors built facilities leading to the marginalization of the local community.

**Figure 2:**
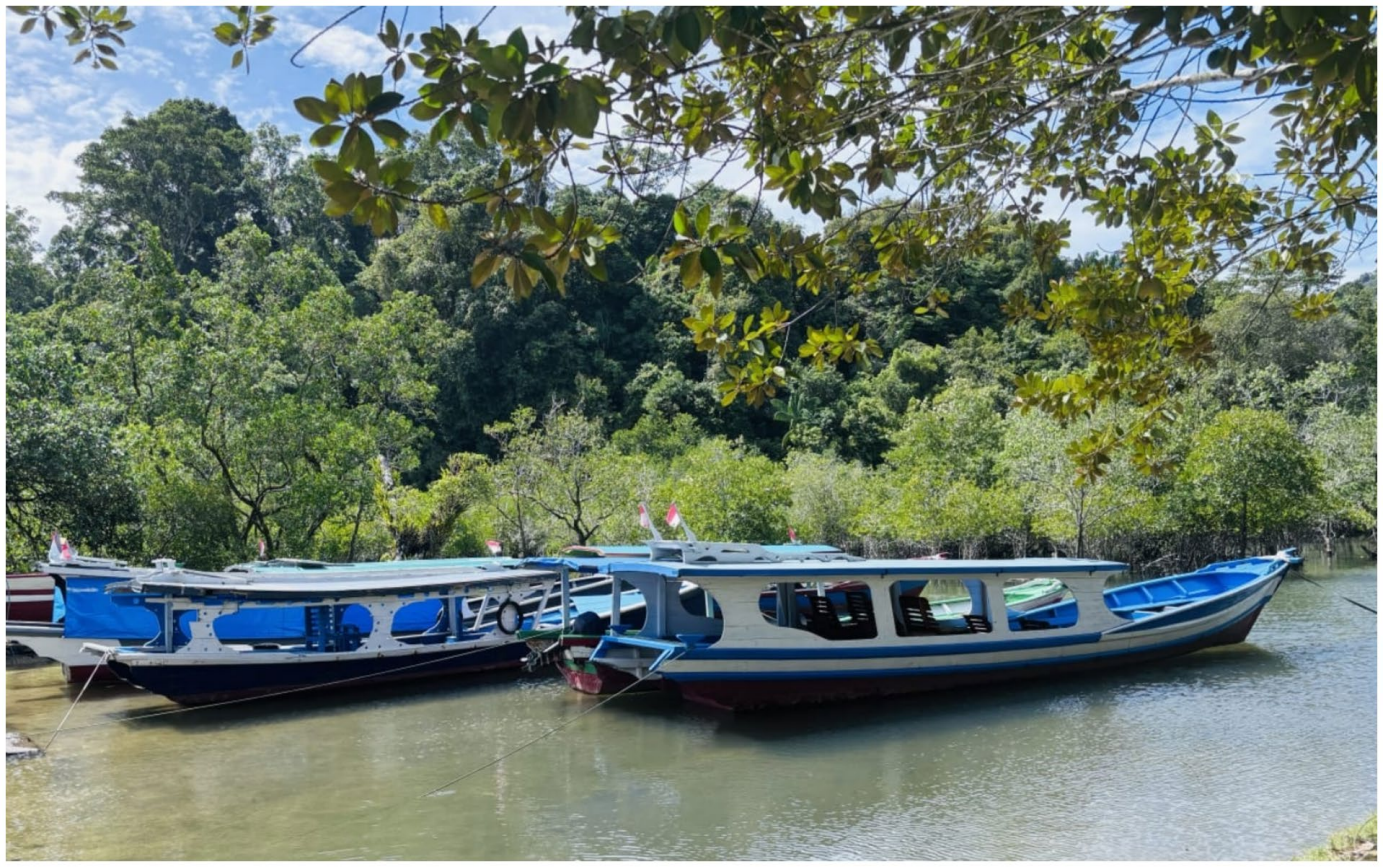
Traditional fishing boat which is also used as tourist transportation to get around the sea and islands.

The participation of the local community in tourism development was evident in various forms of creativity including the establishment of related facilities, using the local nature and culture as the main attractions. This led to the building of diverse tourism facilities independently, under the direction and guidance of the local government, who actively participated in the construction process. Fishing boats were renovated into tourist boats, with the community taking on roles as boat operators and tour guides to cater to tourists’ needs. Furthermore, roughly 100 fishing boats in Mandeh were converted for tourism purposes (Figure 2).

**Figure 3:**
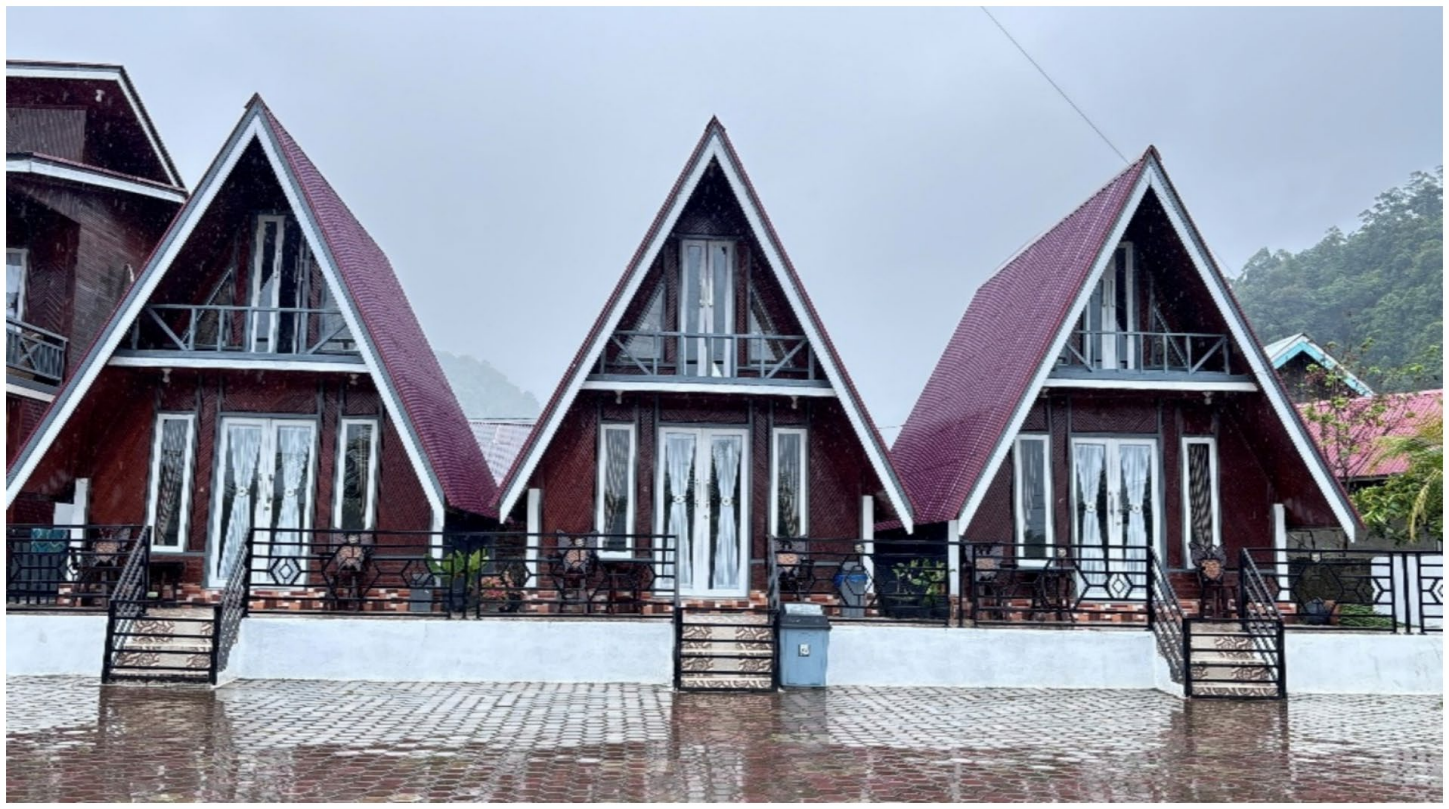
Homestay as a tourist accommodation which is the home of the local community.

Mandeh community offered accommodation services by transforming respective homes into homestays for visiting tourists at relatively affordable prices, in addition to providing tourist transportation (Figure 3). The community also participated in the sale of local creative economy products, including handicrafts made from seafood, regional culinary specialties (such as processed fish and traditional foods), and Mandeh-themed souvenirs, to improve family income.

During holiday season and weekends, tourism sector enabled residents to earn roughly IDR 2,000,000 per day. This shift had a significant positive impact on the economic life, which previously depended solely on uncertain marine resources. Tourism brought new hope to Mandeh community, allowing the evolution from an isolated group reliant on fishing and forest yields. The people became tourism fishermen, offering related services while still preserving respective identity as traditional fishermen.

The transformation in Mandeh area was caused by the internal capacities of the local community. A typical example was the ability to adapt to environmental changes. In this context, adaptation is the ability to survive in difficult conditions, as well as respond to alteration in creative and innovative ways, enabling change to become an opportunity for growth.

Mandeh fishing community, which had lived in isolation for decades, trained the people to continuously learn and adapt to unpredictable natural situations, including changing weather, seasonal fishing, and fluctuating sea conditions. This lengthy experience of adapting to nature became a crucial asset when faced with major changes resulting from the conversion of residential area into tourist destination. The people possessed a high degree of adaptive capacity, which enabled the transformation of challenges posed by the evolution into opportunities for continued survival. The opportunities included the shift in livelihoods from traditional fishing to the provision of tourism services.

The transformation from traditional fishermen to marine tourism operators was not an easy process and required a high degree of adaptability from the local community. This led to the learning of new skills that were completely different from the previous lives, such as serving tourists, communicating with people from diverse backgrounds, managing small businesses, and using digital technology for promotion. However, the adaptation process did not alter the traditional identity and values as fishermen, rather it integrated new knowledge with old values, resulting in a unique tourism form. ZL fisherman who also became tourist boat operator, shared how his friends adapted to the changes that occurred:

“...At first, we thought tourism would interfere with our work as fishermen. This led to worrisome feelings about harming the sea, and the drop in fish stocks because of tourists. But that turned out not to be true, because it was realized that we could still fish and also serve tourists. In the morning, we go out to the sea to fish as always, and in the afternoon or evening, tourists are taken around the island in the same boat. Our fishing boat, used just for fishing, has now been modified into a vessel that can take tourists around the island and the sea...” (Interview on July 17, 2025).

Informants’ accounts showed an interesting adaptation process where communities failed to select between maintaining the new professions, but blended the two. The ability to work as a fisherman in the morning and operate boats in the afternoon or evening showed flexibility in managing time and economic activities. This played an important role in preserving the traditional identity and skills, which had been passed down through generations, while earning extra income from the more lucrative tourism sector. The adaptation occurred in Mandeh community, as the people learned to understand and meet the varying needs of tourists. According to YL, who managed a homestay:

“...I clearly remember the first time tourist stayed at my house, and I was at a loss about what to cook. I only knew how to make fish curry and chili sauce, like we eat every day. It turned out that tourists had different tastes. Then, I also learned to cook other dishes. Now I can cook a variety of dishes..." (Interview, July 18, 2025)

The adaptability of the informants showed the ability to learn new skills. This implied that Mandeh community was open and eager to turn challenges into opportunities to improve respective capabilities. The adaptability was also clear when faced with environmental issues caused by the growing number of tourists. FR, an active member of an environmental awareness group, recounted:

"...During the early years of tourist boom here, we were unprepared for the waste problem. There was a lot of plastic waste from tourists, the beaches were dirty, and the sea polluted. We realized this was a major issue, and if left unchecked, would damage Mandeh's beauty and discourage tourists from returning. We started managing waste, inspiring tourists not to litter the area, by providing trash bins. Currently, many tourists say Mandeh is clean..." (Interview, July 18, 2025)

Based on the explanation of the informant, the community exhibited the ability to respond to evolving problems, and to actively find long-term solutions. Mandeh community’s ability to integrate local knowledge with new tourism needs depicted the characteristics of resilience-based adaptation. This included adaptation that was both reactive and transformational. Moreover, the transformation reflected the dynamics of the social-ecological system, where interactions among humans, the environment, and local institutions unfolded in a structured manner (Folke et al., 2010; Olsson et al., 2014).

In this context, Holling and Gunderson (2002) reported that a resilient social-ecological system could maintain its function while transforming into new, more beneficial structures and practices. This adaptation form was described as a process where the community maintained old practices and also created new, more productive alongside sustainable social, economic, and ecological configurations (Pelling and Manuel-Navarrete, 2011). The transformation was strengthened by adaptive capacity, supported by social capital, community collaboration, and local leadership, which enabled the community to organize collective change (Green, 2005; Bennett et al., 2016).

### 4.2 Family values and solidarity as bonding social capital in marine tourism development

Family values and solidarity were the main social capital that drove the transformation of isolated coastal villages in Mandeh area into sustainable marine tourism destinations. The fishing community had strong social bonds, formed through decades of living together in isolation. In addition, the isolation strengthened the community’s internal solidarity, as its survival depended heavily on mutual cooperation and support. These values were also applied in tourism development, as proven by the statement of HY. The homestay manager in Mandeh stated that:

"...When tourists started arriving in large numbers, we were at a loss for what to do. But since we were used to living together, we agreed to collaborate. Those with boats would take us to the islands, while the people who owned nice houses provided lodging, and individuals who could cook offered food..." (Interview, July 14, 2025)

The solidarity of Mandeh community was further reflected in the flexible yet well-organized role-sharing system. When large numbers of tourists arrived, the people spontaneously divided up the numerous tasks. Some acted as tour guides, others arranged sea transportation, provided food and drinks, as well as organized accommodations. The system operated smoothly because it was based on years of trust and mutual respect.

This division of roles not only enhances the efficiency of tourism services but also functions as a social mechanism to prevent unhealthy competition among local actors. By allocating tasks through mutual agreement and trust, the potential for conflict related to competition for guests, access to resources, and the distribution of economic benefits is minimized. The principle of shared livelihoods and unwritten rules grounded in family-based values operate as instruments of informal social control. These norms constrain individualistic behavior that could otherwise generate social jealousy, inequality, and latent conflict. Accordingly, bonding social capital not only strengthens social cohesion but also serves as a community-based conflict management mechanism, whereby social pressure and moral commitment to the group encourage compliance with collective agreements.

In the context of tourism infrastructure development, collective participation in the form of labor, material contributions, and financial support further reduces the potential for conflict related to costs and responsibilities. This system of voluntary contributions and shared responsibility strengthens a sense of collective ownership of tourism facilities, thereby minimizing unilateral claims and reducing the likelihood of intergroup conflict. AR, a local community leader, added:

"... What is special about us here is that we practically have no conflict in managing tourism industry. The diverse activities are performed peacefully because we have an unwritten rule: sustenance comes from the Almighty and must be shared. We manage tourism industry together because we are a family." (Interview, July 14, 2025)

This value of solidarity was clearly depicted in how collective development issues that came up alongside the growth of tourism industry, were tackled. Tourism infrastructure that needed to be repaired or expanded to support tourists’ comfort, were fixed without waiting for government help. This attitude reflected the independence and confidence of the community in managing changes in the immediate environment. For example, when the dock for tourist boats needed repairs to make it safer and more comfortable for tourists, the entire community contributed both in the form of physical labor, providing building materials namely wood and stone, as well as funds according to each family’s economic capacity. This contribution system was not based on coercion rather on the understanding that tourism infrastructure would benefit the entire community. HD, a former fisherman who worked as tour guide, proudly explained the community’s shared support system.

“...We never see tourists as 'property'. When a guest arrives, the first person to meet them is the one who serves them, because the money made is also for our brothers and sisters. But the income is always shared equally, using our own methods. This is a mutual agreement that does not need to be written down, because everyone understands and knows that we are one big family. Therefore, developing tourism to build the village is our duty...” (Interview on July 14, 2025)

The informant’s explanation above shows that the kinship system is the main foundation for tourism development. The principle of sharing sustenance with fellow boat owners, because of the brotherhood bond is a source of social cohesion created by kinship, shared residence, and destiny. Moreover, shared emotional bonds could unite and bound unwritten commitments. The emotional bond from kinship principles, served as bonding social capital including the foundation for collective awareness and action in tourism development (Hwang and Stewart, 2017; Trinh, 2021). This result was in line with the study by Adger (2003) that shared emotional bonds constituted crucial social capital responsible for uniting communities, and providing a basis for adapting to crisis situations. In this context, bonding social capital mobilized effective collective action in addressing systemic disruptions, such as tourism pressures in coastal areas (Partelow, 2021).

### 4.3 Trust as a basis for participation and cooperation in village development (bridging social capital)

Trust is another form of social capital perceived as a driving factor in the success of tourism development in Mandeh. This attribute is the main foundation for building cooperation between various stakeholders (Raymond, 2006; Soulard et al., 2018). In the framework of social capital theory proposed by Putnam, Coleman, and Fukuyama, it is a fundamental element that enabled the effective collaboration of social actors to achieve common objectives (Coleman, 1990; Dhesi, 2000; Sunkar et al., 2016).

In tourism development, trust did not evolve spontaneously rather it was deeply rooted in local cultural values that had long been part of the social life of the coastal community. This attribute arose from daily social relationships and traditional values that taught warm welcome to guests, trust in others, hospitality, and mutual assistance. The values evolved from the deep understanding of the village’s name, Mandeh, a local Minangkabau word meaning Mother. The name holds a deep philosophical meaning that underlies the behavior of the community. Additionally, because the term means Mother, visiting Mandeh Village implies returning to Mother’s house.

The understanding of the village’s name underpins the local community’s approach to tourists. Based on this perspective, tourists to Mandeh are considered as brothers and sisters who should be treated properly, in a similar manner an individual would be treated at the mother’s house, where comfort, warmth, and trust, are experienced. The value fostered strong trust, which strengthened cooperation in terms of developing tourism that provided tourists with comfort. RL, a local resident, explained how trust was the foundation of running tourism business in Mandeh:

"...We welcome guests here wholeheartedly, because our village is a home for everyone. Mandeh means mother, therefore the village is the home of everyone's mother. Our intention is to make people feel like they are coming home to their mother, because they are our relatives. This is a principle we always adhere to when welcoming guests..." (Interview on July 15, 2025)

Trust in Mandeh community was reflected in the management of homestays and tourist accommodations. Many homeowners rented respective homes as homestays to tourists without a formal booking system or strict deposit requirements. Trust system in Mandeh community also played a crucial role in the development of tourism infrastructure. According to YF, head of tourism awareness group in one of *nagari* areas in Mandeh:

"...When we needed to build a new pier for our tourist boats, money was collected from the community voluntarily. The people with more money contributed more, while those with less fund made donations according to respective means. There was no detailed record of who contributed how much. It was believed everyone contributed according to respective means. The collected funds were also managed by several trusted individuals in the community. Presently, no issues or suspicions about the management of the funds, have been reported..." (Interview, July 15, 2025)

The informant’s statement showed that trust was a fundamental aspect of collective development in Mandeh. In Mandeh community, this attribute extended to relationships with outsiders, including tourists and business people. ED, tour guide, explains how trust was built with tourists:

"...Many tourists visit because they hear from their friends that Mandeh community is trustworthy. This honesty and trustworthiness are what make many tourists come back, bringing their friends along..." (Interview, July 15, 2025)

The explanation of informants proved that trust functioned internally in the community and served as invaluable social capital in building the reputation of tourist destination. This abstract value was put into practice across various aspects of life, including tourism development. In this context, trust has unique characteristics distinguished from other societies, due to the interpersonal nature. It evolved from a philosophy of life passed down through generations. Trust born from Mandeh community’s philosophy of life strengthens internal cohesion, and also functions as bridging social capital, expanding community relationships with actors beyond kinship networks (Raymond, 2006; Soulard et al., 2018).

The bridging of social capital enabled the community to collaborate with various parties, expanding access to economic opportunities, innovation, and resources needed for sustainable tourism development (Henocque, 2013; Nunkoo, 2017). This was consistent with the theory proposed by Robert Putnam, that bridging social capital connected different groups, creating information exchange and collaboration across social boundaries (Putnam, 2015). Considering tourism context, relationships built through trust enhanced destination credibility, and created authentic tourism experiences, alongside factors proven to strengthen tourist loyalty and destination reputation (Nunkoo and Ramkissoon, 2011). Bridging social capital facilitated more inclusive collective agency, which enabled the community to manage external pressures while effectively exploiting new economic opportunities (McGehee et al., 2010).

### 4.4 Collaboration with migrants and stakeholders as linking social capital

Tourism development in Mandeh was driven by the use of social networks, which acted as linking social capital, forming the basis for collaboration with various parties. This included Mandeh migrants and external groups, namely the government, universities, Non-Governmental Organizations (NGOs), environmental conservation, and diving communities. The strength of the social networks allowed for the formation of more open, mutually supportive interaction patterns oriented toward sustainable tourism development (Henocque, 2013).

Community-government relations was developed through inclusive networking patterns. The government did not operate top-down rather actively engaged community leaders, ninik mamak (village heads), youth, and representatives of *nagari* in various tourism development planning and implementation processes. This process strengthened social connectivity between formal structures and the local community, making residents feel respective positions and voices were heard. SY, a community leader who also actively served as a liaison between the local community and government, explained how social networks functioned in tourism development:

“…Our collaboration with the government has been going well. We actively participated in the development process from the very beginning. We have been invited to discuss and plan the establishment of facilities and tourist attractions. Likewise, in tourism development process, we have engaged in various tourism activities, such as serving as tour guides, boat operators, and even providing our homes as homestays for tourists…” (Interview, July 18, 2025)

The information above described how the government served as a support system for tourism development including the local community. In addition, strong social networks were also being built through collaborations with universities, NGOs, and the environmental conservation community. These relationships were develop through coaching activities that increased capacity as tourism service providers and a commitment to marine resource sustainability. The inter-organizational network, enabled Mandeh community to access mentoring, new knowledge, and various empowerment programs. This was outlined by the statement of RN, a member of tourism awareness group, who stated that:

“...With our network, we often received information about aid programs from the government, NGOs, or companies with CSR programs. We also had the contacts of tourism offices, universities, and various institutions who could help develop proposals or provide technical assistance. For example, a university lecturer helped with the creation of a website. Without this network, we might not have known where to start or would have had to pay a lot of money...” (Interview, July 20, 2025).

The information showed that social networks functioned as communication channels and collaborative mechanisms that generated tangible benefits. These included broader flow of information, opportunities for collaboration, a clear distribution of roles, collective commitment to developing tourism and preserving the area.

In Mandeh context, these social networks were not solely driven by external assistance. The power of migrants (Mandeh community who live outside the area) played a strategic role as a bridge between the local community and external networks. These individuals occupied the intersection of *nagari* cultural identity and modern social networks in large cities. Additionally, the migrants were viewed as bringing positive interests and free from exploitative motives, because of the emotional attachment to respective hometowns.

Migrants often acted as bridges between the community and investors, academics, the media, including business networks. The extensive connections, enabled the community gain access to various opportunities, such as digital marketing, tourism package collaborations, promotional support, and even capital assistance for homestay managers and local culinary businesses. Migrant networks also served as a mediator when differing interests arose between the community and investors or the government. These people were perceived as understanding both sides resulting in the ability to reduce potential conflict. The role of migrants in tourism development was evident in the statement of DS, secretary of tourism awareness group in one of the *nagari*, who recounted the diverse experiences in building tourism facilities:

“… Tourists played a crucial role in developing Mandeh tourism. In the early days of the development process, it was the migrants who often connected us with the central government. We even helped build tourist facilities. With this kind of network, development was faster and less burdensome for the local community, whose economic situation was still limited…” (Interview on July 18, 2025)

The importance of the role played by migrants in tourism development was also described by RD:

“…I have worked in tourism sector for a long time. When Mandeh began to develop, I became a liaison between travel agents in the city and Mandeh community. I coordinated with family and friends in the village to provide guides, transportation, homestays, and everything needed by tourists, whenever, they wanted to visit Mandeh.” (Interview on July 19, 2025)

The informants’ statements outlined the important role of migrants in bridging relationships between the local community and external parties. Migrants provided linking social capital, thereby fostering collaboration with stakeholders outside the village. This network expanded community access to information and opportunities as well as strengthened collaborative mechanisms to maintain regional sustainability, enhance local competencies, and expand Mandeh’s connectivity in the broader tourism network.

The linking of social capital expanded access to resources and economic opportunities. This enhanced the community’s adaptive capacity to maintain environmental sustainability (Raymond, 2006; Sunkar et al., 2016), reduce stressors caused by crises (King et al., 2021), and improve community capabilities for developing businesses (Dhesi, 2000; Finkel, 2010). Moreover, vertical social networks were a crucial foundation for the resilience and governance of socio-ecological systems (Adger, 2003).

## 5 Discussion

The results were comprehensively understood through a combination of Talcott Parsons’ Structural Functional, Adaptive Capacity, and Social Capital Theories. In the Structural Functional Theory framework proposed by Parsons through AGIL concept, to achieve equilibrium in the face of change, every social system must fulfill four main functions: Adaptation (A), Goal Attainment (G), Integration (I), and Latency (L) (Parsons, 1961; Robertson and Turner, 1991). In this context, these four functions were fulfilled simultaneously in Mandeh community, ensuring that changes to the village identity did not cause disorganization. The changes strengthened the community’s collective ability to actively participate in tourism development.

Adaptation (A) function was clearly evident in the results regarding the adaptive capacity of Mandeh community. According to this theory, a community’s ability to learn, develop flexibility, and adapt to environmental changes was key to successful social transformation (Adger, 2003; Folke et al., 2010). This was reflected in the way Mandeh community combined traditional fishing activities with new roles as tourism operators, modifying boats into tourist vessels, as well as learning service and business management skills. The adaptation process showed that the community responded reactively, proactively and innovatively, enabling the transformation of external pressures into economic opportunities (Pelling and Manuel-Navarrete, 2011; Cinner et al., 2018; Susilo et al., 2021).

Integration function (I) was carried out through bonding social capital, which included family values, solidarity, and mutual cooperation in marine tourism development. The bonding of social capital helped enhance internal cohesion, reduced conflicts, and established unwritten social norms followed by all members (Putnam, 2015). Meanwhile, social solidarity was derived from living together in isolated conditions for decades, creating a strong collective identity. The bonding of social capital works to maintain internal order, ensured that change did not lead to disintegration, rather it strengthened the existing structure (McGehee et al., 2015; Soulard et al., 2018; Cofré-Bravo et al., 2019).

The function of Goal Attainment (G) was evident in the results on trust as a form of bridging social capital. Trust between individuals and groups was a critical part of social capital because it enabled coordination, lowered transaction costs, and fostered stable, long-term cooperation (Coleman, 1990). The attribute helped the community set collective goals for sustainable tourism development as well as mobilized shared resources for the achievement purposes (Ahn, 2005; Raymond, 2006; Sunkar et al., 2016). The finding supports Parsons’s idea that social goals are achievable when there is strong social coordination. A typical mechanism is trust, a social norm that influences the behavior of community members.

Latency (L) function, or pattern maintenance, was reflected in linking social capital. This vertical network connected Mandeh community with migrants, the government, academics, NGOs, and environmental conservation groups. The linking of social capital offered space and opportunities for the community to access resources, policy legitimacy, increased knowledge, development aid, and regional promotion for sustainable tourism (Yunus et al., 2020). This vertical network helped preserve values and motivation as well as ensured that social transformation continued sustainably with the support of strong external structures (Moscardo, 2014; Cofré-Bravo et al., 2019).

The analysis showed that the rapid transformation in Mandeh was due to the dynamic interaction between the community’s adaptive capacity and strength of its social capital. Furthermore, adaptation enabled the community to seize new opportunities (Holling and Gunderson, 2002; Folke et al., 2003), internal solidarity maintained social cohesion (Sunkar et al., 2016; Hwang and Stewart, 2017; Rodriguez-Giron and Vanneste, 2019), trust facilitated collaboration (Kramer et al., 1996; Ahn, 2005; Raymond, 2006), and vertical networks provided access to external resources (Cofré-Bravo et al., 2019). The synergy of these factors explained why significant changes in Mandeh happened quickly while still preserving local social and cultural identity.

Although this transformation process has the potential to generate conflict including competition among local actors, unequal access to tourism benefits, and shifts in power relations within communities these tensions can be managed within the AGIL framework through the integration (I) and latency (L) functions strengthened by social capital (Robertson and Turner, 1991; Schwandt, 2010). Solidarity, social norms, and deliberative practices function as instruments of informal social control that constrain conflict escalation (Rocca and Zielinski, 2022), while vertical networks provide spaces for mediation, legitimacy, and institutional support (Coleman, 1990). Consequently, social capital and adaptive capacity not only drive transformation but also operate as social buffering mechanisms that maintain system stability, enabling change to unfold in a relatively harmonious, inclusive, and sustainable manner (Dhesi, 2000; Adger, 2003; McGehee et al., 2010).

## 6 Conclusion

In conclusion, this study showed that adaptability and social capital were essential for the successful development of marine tourism villages in Mandeh *nagari*. Adaptive capacity served as the main foundation that motivated the transformation of the community, while maintaining its social and cultural identity. Simultaneously, social capital underpined collective action, motivating participation and collaboration in village development. Furthermore, social capital operates as a key mechanism for managing and mitigating potential conflicts associated with tourism development, including competition, unequal benefit distribution, and shifts in local power relations. Through shared norms, trust, and deliberative practices, the community is able to contain social tensions and maintain cohesion, thereby supporting a more harmonious, inclusive, and sustainable transformation process. Both adaptive capacity and social capital originated from sociocultural values inherited from ancestors, a society that once lived in isolation to withstand changing environmental conditions. These values were also applied when the area evolved into tourist destination. The transformation of Mandeh into a marine tourism center proved that sustainable marine tourism development only occurred supposing the local community, including fishermen, were actively engaged as major stakeholders.

The implications of this study enhanced the literature on community-based tourism and provided an important contribution to the development models that were equitable, resilient, and focused on socio-ecological sustainability. Despite proving that adaptive capacity and social capital were the main factors in transforming isolated villages into marine tourism centers, it failed to examine in detail how local actors such as traditional leaders, youth, women, business groups, and migrants served as agents of change, influencing the direction, pace, and dynamics of this transformation. In addition, this study did not sufficiently examine the dynamics of conflict and power relations that may emerge alongside tourism development. Due to the limitations, future research is encouraged to more systematically investigate the roles of local actors as agents of change, including the strategies, leadership practices, negotiations, and power dynamics through which communities are mobilized and conflicts are managed during tourism-driven transformation processes.

## Data Availability

The raw data supporting the conclusions of this article will be made available by the authors, without undue reservation.

## References

[ref1] Adger W. N. (2003). Social capital, collective action, and adaptation to climate changes. Econ. Geogr. 79:4.

[ref2] Ahn T.K.. (2005) ‘Trust and collective action: concepts and causalities’, in Annual Meeting of the American Political Science Association, August 28–September 1, p. 28.

[ref3] Alhadi Z. Muchtar B. Evanita S. (2023). Developing a community-based tourism model for sustainable tourism in the Mandeh Area, West Sumatra Province, Indonesia. Int. J. Sustain. Dev. Plann. 18, 1166–1174. doi: 10.18280/ijsdp.181114

[ref4] Apps K. Dimmock K. Huveneers C. (2018). Turning wildlife experiences into conservation action: can white shark cage-dive tourism influence conservation behaviour? Mar. Policy 88, 108–115. doi: 10.1016/j.marpol.2017.11.024

[ref5] Atasoy F. (2021). “Authenticity in tourism” in Academic Turkish World Studies: Tourism, Culture, Art and Architecture. ed. F. Turkmen (Berlin: Peter Lang), 377–398.

[ref6] Bennett N. J. Blythe J. Tyler S. Ban N. C. (2016). Communities and change in the anthropocene: understanding social-ecological vulnerability and planning adaptations to multiple interacting exposures. Reg. Environ. Chang. 16, 907–926. doi: 10.1007/s10113-015-0839-5

[ref7] Biro Komunikasi Publik Kementrian PUPR (2019). Kementerian PUPR Rampungkan 41,08 Km Jalan Akses Kawasan Wisata Mandeh. Jakarta: Republika.

[ref8] Booth H. Mourato S. Milner-Gulland E. J. (2022). Investigating acceptance of marine tourism levies, to cover the opportunity costs of conservation for coastal communities. Ecol. Econ. 201:107578. doi: 10.1016/j.ecolecon.2022.107578

[ref9] BPS Kabupaten Pesisir Selatan (2025). Pesisir Selatan Painan Available online at: https://pesselkab.bps.go.id/id/publication/2025/02/28/8c453966c49d9e69aed04ef3/kabupaten-pesisir-selatan-dalam-angka-2025.html (Accessed August 25, 2025).

[ref10] Cinner J. E. Adger W. N. Allison E. H. Barnes M. L. Brown K. Cohen P. J. . (2018). Building adaptive capacity to climate change in tropical coastal communities. Nat. Clim. Chang. 8, 117–123. doi: 10.1038/s41558-017-0065-x

[ref11] Cofré-Bravo G. Klerkx L. Engler A. (2019). Combinations of bonding, bridging, and linking social capital for farm innovation: how farmers configure different support networks. J. Rural. Stud. 69, 53–64. doi: 10.1016/j.jrurstud.2019.04.004

[ref12] Coleman J. S. (1990). Social capital. Found. Soc. Theory 2, 300–321.

[ref13] Cong L. C. Van Chi T. T. (2021). The sustainability of marine tourism development in the South Central Coast, Vietnam. Tour. Plan. Dev. 18, 630–648. doi: 10.1080/21568316.2020.1837226

[ref14] Croes R. Lee S. H. Olson E. D. (2013). Authenticity in tourism in small island destinations: a local perspective. J. Tour. Cult. Chang. 11, 1–20. doi: 10.1080/14766825.2012.759584

[ref15] Denzin N. K. Lincoln Y. S. (2009). Handbook of Qualitaive Research. Yogyakarta: Pustaka Pelajar.

[ref16] Dhesi A. S. (2000). Social capital and community development. Community Dev. J. 35, 199–214. doi: 10.1093/cdj/35.3.199

[ref17] Fatimah S. Ramadhan D. (2019). Sustainable tourism integrated tourism area based on culture and local wisdom at Mandeh Area. Int. J. Tour. Herit. Recreat. Sport 1, 1–7. doi: 10.24036/ijthrs.v1i1.14

[ref18] Fernandez-Abila C. J. Tan R. Dumpit D. Z. Gelvezon R. P. Arcala Hall R. Lizada J. . (2024). Characterizing the sustainable tourism development of small islands in the Visayas, Philippines. Land Use Policy 137:106996. doi: 10.1016/j.landusepol.2023.106996

[ref19] Finkel R. (2010). “Dancing around the ring of fire”: Social capital, tourism resistance, and gender dichotomies at Up Helly Aa in Lerwick, Shetland. Event Manag. 14, 275–285. doi: 10.3727/152599510X12901814778023

[ref20] Folke C. Colding J. Berkes F. (2003). Synthesis: Building resilience and adaptive capacity in social-ecological systems, in: Navigating Social-Ecological Systems: Building Resilience for Complexity and Change. eds. Berkes F. Colding J. Folke C. (Cambridge: Cambridge University Press). pp. 352–387.

[ref21] Folke C. Carpenter S. R. Walker B. Scheffer M. Chapin T. Rockström J. (2010). Resilience thinking: integrating resilience, adaptability and transformability. Ecol. Soc. 15, 1–9.

[ref22] Fonseca C. da Silva C. P. Calado H. Moniz F. Bragagnolo C. Gil A. . (2014). Coastal and marine protected areas as key elements for tourism in small islands. J. Coast. Res. 70, 461–466. doi: 10.2112/SI70-078.1

[ref23] Green R. (2005). Community perceptions of environmental and social change and tourism development on the island of Koh Samui, Thailand. J. Environ. Psychol. 25, 37–56. doi: 10.1016/j.jenvp.2004.09.007

[ref24] Hall C. M. (2001). Trends in ocean and coastal tourism: the end of the last frontier? Ocean Coast. Manage. 44, 601–618. doi: 10.1016/S0964-5691(01)00071-0

[ref25] Hampton M. P. Jeyacheya J. (2015). Power, ownership and tourism in small islands: evidence from Indonesia. World Dev. 70, 481–495. doi: 10.1016/j.worlddev.2014.12.007

[ref26] Henocque Y. (2013). Enhancing social capital for sustainable coastal development: is satoumi the answer? Estuar. Coast. Shelf Sci. 116, 66–73. doi: 10.1016/j.ecss.2012.08.024

[ref27] Hofman K. Walters G. Hughes K. (2022). The effectiveness of virtual vs real-life marine tourism experiences in encouraging conservation behaviour. J. Sustain. Tour. 30, 742–766. doi: 10.1080/09669582.2021.1884690

[ref28] Holling C. S. Gunderson L. H. (2002). Resilience and adaptive cycles. In: Panarchy: Understanding transformations in systems of humans and nature. eds. Gunderson L. H. Holling C. S. (Washington: Island Press). 25–62.

[ref29] Hwang D. Stewart W. P. (2017). Social capital and collective action in rural tourism. J. Travel Res. 56, 81–93. doi: 10.1177/0047287515625128

[ref30] Khakzad S. Griffith D. (2016). The role of fishing material culture in communities’ sense of place as an added-value in management of coastal areas. J. Mar. Isl. Cult. 5, 95–117. doi: 10.1016/j.imic.2016.09.002

[ref31] King C. Iba W. Clifton J. (2021). Reimagining resilience: COVID-19 and marine tourism in Indonesia. Curr. Issue Tour. 24, 2784–2800. doi: 10.1080/13683500.2021.1873920

[ref32] Kinseng R. A. Nasdian F. T. Fatchiya A. Mahmud A. Stanford R. J. (2018). Marine-tourism development on a small island in Indonesia: blessing or curse? Asia Pac. J. Tour. Res. 23, 1062–1072. doi: 10.1080/10941665.2018.1515781

[ref33] Kong M. H. (2024). Marine Cultural Tourism Gwangalli Eobang Festival: Cultural Inheritance and Efficiency Enhancement from a Humanistic Perspective. J. Marine Island Cult. 13, 111–134. doi: 10.21463/jmic.2024.13.1.07

[ref34] Kramer R. M. Brewer M. B. Hanna B. A. (1996). Collective Trust and Collective Action, Trust in Organizations: Frontiers of Theory and Research. Thousand Oaks CA: Sage Publications.

[ref35] Loulanskia T. Loulanski V. (2011). The sustainable integration of cultural heritage and tourism: a meta-study. J. Sustain. Tour. 19, 837–862. doi: 10.1080/09669582.2011.553286

[ref36] Mangubhai S. Sykes H. Manley M. Vukikomoala K. Beattie M. (2019). Contributions of tourism-based Marine Conservation Agreements to natural resource management in Fiji. Ecol. Econ. 171:106607. doi: 10.1016/j.ecolecon.2020.106607

[ref37] McGehee N. G. Knollenberg W. Komorowski A. (2015). The central role of leadership in rural tourism development: a theoretical framework and case studies. J. Sustain. Tour. 23, 1277–1297. doi: 10.1080/09669582.2015.1019514

[ref38] McGehee N. G. Lee S. O’Bannon T. L. Perdue R. R. (2010). Tourism-related social capital and its relationship with other forms of capital: An exploratory study. J. Travel Res. 49, 486–500. doi: 10.1177/0047287509349271

[ref39] Misra M. Miller M. L. (2022). Marine conservation tourism and the Giant Pacific Octopus: A SWOT analysis of two public engagement programs and the viability of a hybrid program at the Seattle Aquarium Washington, USA. Reg. Stud. Mar. Sci. 52:102231.

[ref40] Moscardo G. (2014). Tourism and community leadership in rural regions: linking mobility, entrepreneurship, tourism development and community well-being. Tour. Plan. Dev. 11, 354–370. doi: 10.1080/21568316.2014.890129

[ref41] Mura P. Tavakoli R. (2014). Tourism and social capital in Malaysia. Curr. Issues Tour. 17, 28–45. doi: 10.1080/13683500.2012.718320

[ref42] Ning W. (2017). Rethinking authenticity in tourism experience. Polit. Nat. Cult. Herit. Tour. 26, 469–490.

[ref43] Nunkoo R. (2017). Governance and sustainable tourism: what is the role of trust, power and social capital? J. Destin. Mark. Manag. 6, 277–285. doi: 10.1016/j.jdmm.2017.10.003

[ref44] Nunkoo R. Ramkissoon H. (2011). Developing a community support model for tourism. Ann. Tour. Res. 38, 964–988. doi: 10.1016/j.annals.2011.01.017

[ref45] Olsson P. Galaz V. Boonstra W. J. (2014). Sustainability transformations: a resilience perspective. Ecol. Soc. 19, 1–13. doi: 10.5751/ES-06799-190401

[ref46] Orams M. (2002). Marine Tourism: Development, Impacts and Management. London: Routledge.

[ref47] Papageorgiou M. (2016). Coastal and marine tourism: a challenging factor in Marine Spatial Planning. Ocean Coast. Manag. 129, 44–48. doi: 10.1016/j.ocecoaman.2016.05.006

[ref48] Parsons T. (1961). Some considerations on the theory of social change. Rural. Sociol. 26:219.

[ref49] Partelow S. (2021). Social capital and community disaster resilience: post-earthquake tourism recovery on Gili Trawangan, Indonesia. Sustain. Sci. 16, 203–220. doi: 10.1007/s11625-020-00854-2 32901208 PMC7471487

[ref50] Pelling M. Manuel-Navarrete D. (2011). From resilience to transformation: the adaptive cycle in two Mexican urban centers. Ecol. Soc. 16, 1–11.

[ref51] Praptiwi R. A. Maharja C. Fortnam M. Chaigneau T. Evans L. Garniati L. . (2021). Tourism-based alternative livelihoods for small island communities transitioning towards a blue economy. Sustainability 13:6655. doi: 10.3390/su13126655

[ref52] Pretty J. Ward H. (2001). Social capital and the environment. World Dev. 29, 209–227. doi: 10.1016/s0305-750x(00)00098-x

[ref53] Putnam R. D. (2015). Bowling Alone: America’s Declining Social Capital The City Reader. London: Routledge, 188–196.

[ref54] Rahman M. K. Masud M. M. Akhtar R. Hossain M. M. (2022). Impact of community participation on sustainable development of marine protected areas: assessment of ecotourism development. Int. J. Tour. Res. 24, 33–43. doi: 10.1002/jtr.2480

[ref55] Raymond L. (2006). Cooperation without trust: overcoming collective action barriers to endangered species protection. Policy Stud. J. 34, 37–57. doi: 10.1111/j.1541-0072.2006.00144.x.

[ref56] Reddy K. Sailesh B. (2024). Integrating marine tourism into the blue economy framework. J. Environ. Manag. Tour. 15, 501–520. doi: 10.14505/jemt

[ref57] Robertson R. Turner B. S. (1991). “An introduction to Talcott Parsons: theory, politics and humanity” in Talcott Parsons: Theorist of Modernity. eds. Robertson R. Turner B. S. (London: Sage Publication).

[ref58] Rocca L. H. D. Zielinski S. (2022). Community-based tourism, social capital, and governance of post-conflict rural tourism destinations: the case of Minca, Sierra Nevada de Santa Marta, Colombia. Tour. Manag. Perspect. 43:100985. doi: 10.1016/j.tmp.2022.100985.

[ref59] Rodriguez-Giron S. Vanneste D. (2019). Social capital at the tourist destination level: determining the dimensions to assess and improve collective action in tourism. Tour. Stud. 19, 23–42. doi: 10.1177/1468797618790109.

[ref60] Schwandt D. (2010). “Collective learning as social change: Integrating complex adaptive systems and structuration with Parsons theory of action” in Essays in Honour of Talcott Parsons, (Poynton, Cheshire, UK: Midrash) 125–150.

[ref61] Sharpley R. Ussi M. (2014). Tourism and governance in small island developing states (SIDS): the case of Zanzibar. Int. J. Tour. Res. 16, 87–96. doi: 10.1002/jtr.1904

[ref63] Soulard J. Knollenberg W. Boley B. B. Perdue R. R. McGehee N. G. (2018). Social capital and destination strategic planning. Tour. Manag. 69, 189–200. doi: 10.1016/j.tourman.2018.06.011

[ref64] Srinivasan M. Kaullysing D. Bhagooli R. Pratt S. (2022). “Marine tourism and the blue economy: perspectives from the Mascarene and Pacific islands” in Blue Economy: An Ocean Science Perspective. eds. Urban Jr E. R. Ittekkot V. (Singapore: Springer Nature Singapore). doi: 10.1007/978-981-19-5065-0_6

[ref65] Sunkar A. Meilani R. Rahayuningsih T. Muntasib E. K. S. H. (2016). Social capital: a basis for community participation in fostering environmental education and the heritage tourism development of Cibalay Megalithic Site. E-J. Tour. 3, 120–129. doi: 10.24922/eot.v3i2.25256

[ref66] Susilo E. Purwanti P. Fattah M. Qurrata V. A. Narmaditya B. S. (2021). Adaptive coping strategies towards seasonal change impacts: Indonesian small-scale fisherman household. Heliyon 7:e06919. doi: 10.1016/j.heliyon.2021.e06919, 33997422 PMC8105632

[ref67] Tegar D. Saut Gurning R. O. (2018). Development of marine and coastal tourism based on blue economy. Int. J. Mar. Eng. Innov. Res. 2, 128–132. doi: 10.12962/j25481479.v2i2.3650

[ref68] Trinh T. T. (2021). Social capital and residents’ participation to rural community-based tourism development: an initial exploratory study in North Central Coastal Vietnam. J. Tour. Sustain. 5, 36–43. Available online at: https://journal.ontourism.academy/index.php/jots/article/view/109

[ref69] Wang L. Zhang H. (2019). The impact of marine tourism resources development on sustainable development of marine economy. J. Coast. Res. 94, 589–592.

[ref70] Wilson C. Tisdell C. (2003). Conservation and economic benefits of wildlife-based marine tourism: sea turtles and whales as case studies. Hum. Dimens. Wildl. 8, 49–58. doi: 10.1080/10871200390180145

[ref71] Xie P. F. Chandra V. Gu K. (2013). Morphological changes of coastal tourism: a case study of Denarau Island, Fiji. Tour. Manag. Perspect. 5, 75–83. doi: 10.1016/j.tmp.2012.09.002

[ref72] Yunus S. Zainal S. Jalil F. (2020). Social network, trust, and collective action of Aceh. Int. J. Psychosoc. Rehabil. 24, 184–192.

[ref73] Zamzami L. (2021). The effect of ecotourism development on marine conservation are in West Sumatra, Indonesia. Geo J. Tour. Geosites 38, 1166–1174. doi: 10.30892/gtg.38423-757

